# Enhancing Nutritional Profile of Pasta: The Impact of Sprouted Pseudocereals and Cushuro on Digestibility and Health Potential

**DOI:** 10.3390/foods12244395

**Published:** 2023-12-06

**Authors:** Luz María Paucar-Menacho, Juan Carlos Vásquez Guzmán, Wilson Daniel Simpalo-Lopez, Williams Esteward Castillo-Martínez, Cristina Martínez-Villaluenga

**Affiliations:** 1 Departamento Académico de Agroindustria y Agronomía, Facultad de Ingeniería, Universidad Nacional del Santa, Nuevo Chimbote 02712, Peru; luzpaucar@uns.edu.pe (L.M.P.-M.); wsimpalol@uns.edu.pe (W.D.S.-L.); wcastillo@uns.edu.pe (W.E.C.-M.); 2 Department of Technological Processes and Biotechnology, Institute of Food Science, Technology and Nutrition (ICTAN), Spanish National Research Council (CSIC), 28040 Madrid, Spain

**Keywords:** sprouted pseudocereals, formulation, pasta, nutritional quality, bioactive compounds, digestion

## Abstract

We hypothesized that optimizing the formulation of pasta by incorporating sprouted pseudocereal flours, specifically quinoa (*Chenopodium quinoa* Willd) or kiwicha (*Amaranthus caudatus* L.) and cushuro (*Nostoc sphaericum* Vaucher ex Bornet & Flahault) flours, could offer the potential to simultaneously enhance nutritional quality and health-promoting properties in pasta. In this study, our objective was to optimize the formulation of composite flour (a ternary blend of wheat, sprouted pseudocereal, and cushuro flours) using a mixture composite design to maximize total soluble phenolic compounds (TSPC), γ-aminobutyric acid (GABA), antioxidant activity, and mineral bioaccesilability by reducing phytic acid (PA) content. Two optimal formulations were identified: one consisting of 79% wheat flour (WF), 13% SQF, and 8% CuF (oPQC), and the other composed of 70% WF, 15% SKF, and 15% CuF (oPKC). These optimized pastas exhibited reduced starch content and notably higher levels of total dietary fiber (1.5–3.61-fold), protein (1.16-fold), fat (1.3–1.5-fold), ash (2.2–2.7-fold), minerals (K, Na, Fe, Zn, Mg, Mn, and Ca), PA (3–4.5-fold), TSPC (1.3–1.9-fold), GABA (1.2–2.6-fold), and ORAC (6.5–8.7-fold) compared to control pasta (100% WF). Notably, the glycemic index of oPQC (59.8) was lower than that of oPKC (54.7) and control pasta (63.1). The nutritional profile of the optimized pasta was largely retained after cooking, although some significant losses were observed for soluble dietary fiber (18.2–44.0%), K (47.5–50.7%), Na (42.5–63.6), GABA (41.68–51.4%), TSPC (8–18%), and antioxidant activity (45.4–46.4%). In vitro digestion of cooked oPQC and oPKC demonstrated higher bioaccessible content of GABA (6.7–16.26 mg/100 g), TSPC (257.7–261.8 mg GAE/100 g), Ca (58.40–93.5 mg/100 g), and Fe (7.35–7.52 mg/100 g), as well as antioxidant activity (164.9–171.1 µmol TE/g) in intestinal digestates compared to control pasta. These findings suggest that the incorporation of sprouted pseudocereals and cushuro flour offers a promising approach to enhance the nutritional quality and bioactive content of wheat-based pasta, potentially providing health benefits beyond traditional formulations.

## 1. Introduction

Pasta is a staple food worldwide that is highly consumed due to its pleasant sensory properties, low cost, and ease of preparation. Conventional durum wheat-based recipes exhibit a poor nutritional profile, being low in proteins, dietary fibers (DF), and micronutrients [1]. Furthermore, these products typically have a high glycemic index (GI) due to the high content in rapidly digested carbohydrates [2]. Currently, the emphasis in nutritional science has transitioned to the idea of optimal nutrition, striving to optimize daily diets in terms of nutrients and health-promoting phytochemicals. Within this context, new formulations of functional pasta have been suggested, and there has been a push for innovation in pasta production. The various examples of product innovation on the market include wholegrain, multigrain, gluten-free, and pulse- and vegetable-enriched pasta [3].

Within this context, pasta formulation using composite flours is a field of intense investigation. For instance, blends of semolina/wheat flour and pseudocereal flours richer in nutrients and phytochemicals have been widely explored in pasta making to enhance nutritional profile [4]. Andean pseudocereal grains such as quinoa (*Chenopodium quinoa* Willd.) and kiwicha (*Amaranthus caudatus* L.) are characterized by a low GI, high-quality protein, and high content in DF, essential minerals, vitamins, essential amino acids, and unsaturated fatty acids [4,5]. They also possess elevated levels of polyphenols with commendable antioxidant properties [4]. Specifically, various proportions of amaranth flour (35%, 50%, 55%, and 70%) were utilized as substitutes for semolina in pasta preparation [6]. Regardless of the substitution levels, amaranth pasta exhibited a total polyphenol content exceeding that of 100% semolina pasta (0.98 mg of ferulic acid equivalents/g dm), with values ranging from 1.54 to 3.37 mg ferulic acid equivalents/g dm [6]. On the other hand, macro- and microalgae flours are raw materials being investigated as functional ingredients for the development of innovative pasta products. The Andean microalgae *Nostoc sphaericum* Vaucher ex Bornet & Flahault is a promising raw material to increase the nutritional value and overall quality of fresh pasta because of its high content in protein, DF, vitamins (B_1_, B_2_, B_5,_ and B_8_), and minerals (Ca, Fe, Na, and K) [7,8]. This microalga is found in the Andean foothills at altitudes above 3000 m, forming macrocolonies referred to as cushuro. This colonies can be manually harvested, sun-dried, and subsequently sold in local markets [8]. While still a staple in popular or rural cuisine in certain South American regions, this macroalgae has recently been rediscovered for its potential as an ingredient in restaurant menus or for development of healthier products in the food industry, offering cost-effective alternatives [9].

Germination is a strategy enabling the improvement of the nutritional value and phytochemical content of grains and pasta products derived thereof. Sprouted pseudocereal grains have found application as ingredients in several food product categories, not only because of their higher nutritional value, but also for the interesting sensory attributes and technological properties as compared to ungerminated grains. Germination of pseudocereal grains increases the content and availability of nutrients [10,11,12], reduces the levels of antinutritional factors [13,14], and has led to increased amounts of bioactive compounds (phenolic compounds), γ-aminobutyric acid (GABA), and antioxidant activity [10,15,16].

We hypothesized that optimizing the formulation of pasta by incorporating sprouted pseudocereal flours and cushuro could offer the potential to simultaneously enhance nutritional quality, health-promoting properties, and environmental sustainability in pasta production. Furthermore, this optimization aimed to reduce starch digestibility in pasta products and enhance pasta functionality by increasing the bioaccessibility of phenolic compounds, leading to an improved antioxidant effect during gastrointestinal transit.

In this study, our objective was to optimize the formulation of composite flour (a ternary blend of wheat, sprouted pseudocereal, and cushuro flours) to enhance the nutritional and potential health benefits of pasta consumption. The optimization of composite flour formulation was specifically tailored to maximize the content of phenolic compounds, GABA, antioxidant activity, and mineral bioavailability by reducing phytic acid (PA) content. We also assessed changes in nutritional composition, bioactive compounds, and antioxidant activity of the pasta after cooking. Finally, we investigated the impact of optimizing pasta formulation on carbohydrate digestion and the bioaccessibility of Fe, Ca, GABA, and phenolic compounds.

## 2. Materials and Methods

### 2.1. Materials

Two different pseudocereal grains were included in this study: quinoa (*Chenopodium quinoa* Willd.) and kiwicha (*Amaranthus caudatus* L.). These grains were supplied by the Cereals and Native Grains Program (Lima, Peru). Cushuro (*Nostoc sphaericum* Vaucher ex Bornet & Flahault) was purchased in a local market (Chimbote, Perú). Quinoa and kiwicha grains were sprouted at optimal temperature and time to maximize total soluble phenolic compounds (TSPC), GABA, and antioxidant activity as reported previously [11,12]. Sprouted grains and cushuro were dried in a climatic chamber at 60 °C for 24 h. Dried sprouts and cushuro were milled in a MDNT-60XL grinding module and passed through a sieve of 0.20 mm pore size (Torrh, Jarcon del Peru S.R.L., Junín, Peru). Three types of flour were obtained [sprouted quinoa flour (SQF), sprouted kiwicha flour (SKF) and cushuro flour (CuF)] and stored at 4 °C under vacuum in plastic bags. Commercial durum wheat flour (WF, Nicolini, Alicorp S.A.) was purchased in the market (Lima, Peru).

Chemical reagents and enzymes were purchased from Merck KGaA (Darmstadt, Germany), except for the Total Starch, Rapid Integrated Total Dietary Fiber, and Phytic Acid (Phytate)/Total Phosphorus, and D-glucose Assay kits purchased from Megazyme (Wicklow, Ireland). 9-fluorenylmethoxycarbonyl chloride and ortho-phthaldialdehyde reagents were supplied from Agilent (Santa Clara, CA, USA).

### 2.2. Pasta Making

To produce pasta, two types of composite flour were studied: composite flour 1 (blend of WF, SQF, and CuF) and composite flour 2 (blend of WF, SKF, and CuF). One hundred percent refined wheat flour was used to prepare the reference pasta (control). From here on, pasta samples made from composite flour 1, composite flour 2, and refined WF will be referred to PQC, PKC, and C.

For optimization of composite flour formulation, 14 recipes were prepared for partial substitution of WF with different ratios of SQF and CuF (PQC, Figure S1, Table S1) and SKF and CuF (PKC, Figure S2, Table S1) according to a simplex centroid mixture design. For dough preparation (300 g), 180 g of composite flour was mixed with 60 g of hen eggs and 60 g of water. Doughs were kneaded for 10 min and 100 rpm in a mixing bowl and rested in a plastic bag for 30 min. The obtained doughs were extruded and cut as fettuccine using a Pastaia 2 extruder (Italvisa Maquinas LTDA, Tatui, Brazil). Fresh pasta was dried on trays using natural air convection (Falc Oven Model STE-F 52, Treviglio, Italy) at 55 °C until a moisture level of 11% was reached. Dried pasta was stored at 4 °C in vacuum-sealed plastic bags.

### 2.3. Simplex Centroid Mixture Design

For pasta making, composite flour formulation was optimized using the simplex centroid mixture design including three ingredients: WF (A), SQF/SKF (B), and CuF (C), as independent variables. In an experiment with q components, the proportions of the ingredients may be denoted by *x*_1_, *x*_2_, …, *x_q_*, where *x_i_ ≥* 0 for *i =* 1, 2, …, *q* and *∑q_i_ =* 1*x_i_* = 1, where *x_i_* represents the proportion of the i-th component. This equation removes a degree of freedom from the proportions, and the factor space is therefore a (q − 1)-dimensional regular simplex [17]. The run conditions of the factorial design in terms of the experimental conditions and coded values are shown in Table S1.

The design enabled us to approximate the experimental data (Yobs) with a response surface model represented in Equations (1)–(3):

Linear ŷ = ∑q_i_ = 1β_i_x_i_, (1)

Quadratic ŷ = ∑q_i_ = 1β_i_x_i_ + ∑_q−1_^i < j^∑q_j_β_ij_x_i_x_j_, (2)

Special cubic ŷ = ∑q_i_ = 1β_i_x_i_ + ∑_q−1_^i^^< j^∑q_j_β_ij_x_i_x_j_ + ∑_q−2_^i^^< j^∑_q−1_^j^^< k^∑q_k_β_ij_kx_i_x_j_x_k_, (3)

The parameter β_i_ represents the expected response to the pure blend *x_i_* = 1 and *x_j_* = 0 when *j ≠ i*. The term *∑q_i_ =* 1*β_i_x_i_* represents the linear blending portion. When curvature arises from nonlinear blending between component pairs, the parameters β*ij*, which represent either synergistic or antagonistic blending, will be different from zero.

The difference between the experimental data (Y_obs_) and model (Y_calc_) gives the residual (ε). For each response, the R^2^ (squared correlation coefficient) was calculated, which is the fraction of variation of the response explained by the model. For this work, a coefficient of determination higher than 0.8, a significance level < 0.10, and a ratio F_cal_/F_tab_ ≥ 3.0 were adopted to ensure a good prediction of the mathematical models, without lack of fit. The response variables were PA, GABA, TSPC, and ORAC. The desirability function was used to optimize response variables within a desired range. Level of importance and an objective (maximize, minimize, or keep range) was assigned to each response variable. The experimental data were analyzed with the statistical package Design-Expert V.11.0.1 (Stat-Ease Inc., Minneapolis, MN, USA). Design-Expert was used to solve the second-order polynomial regression equation.

### 2.4. Nutritional Composition

Proximate composition, including moisture, protein, fat, and ash content of flours and pasta (raw and cooked) was determined using the official methods AACC 44–15A, AACC 46–13, AACC 30–10, AACC 08–03, respectively (AACC, 2000). Starch content was measured using the enzymatic total starch assay kit (K-TSTA-100A). Total dietary fiber (TDF) and their soluble (SDF) and insoluble (IDF) fractions were determined using the rapid integrated total dietary fiber (K-RINTDF) assay kit. Data were expressed as g/100 g of dry weight (dw).

Mineral content in flours, pasta, and digestates was determined by atomic absorption using an Analytikjena ContrAA 700 high-resolution atomic absorption spectrometer (Analytik Jena AG, Jena, Germany) equipped with a Xenon short-arc lamp (GLE, Berlin, Germany) operating in a “hot spot” mode as the radiation source. An air–acetylene flame was used for determination of K, Na, Fe, Zn, Mg, and Mn, and a fuel-rich nitrous oxideacetylene flame was used for Ca. Calibration was carried out using aqueous standards, and determinations were performed at the main atomic lines for K (766.5 nm), Na (589.0 nm), Fe (248.3 nm), Zn (213.9 nm), Mg (285.2 nm), Mn (279.5 nm) and Ca (422.7 nm). Data were expressed as mg/100 g dw.

### 2.5. Phytic Acid (PA)

PA content was quantified using the Phytic Acid (Phytate)/Total Phosphorus Assay kit (K-PHYT), and results were expressed in g/100 g dw.

### 2.6. Total Soluble Phenolic Compounds (TSPC)

TSPCs were determined in flours, pasta, and digestates by the fast blue BB reaction, as described previously [18]. Briefly, 50–100 mg of sample was extracted with 1 mL of 80% methanol in 0.1% formic acid. Samples were placed on a Thermomixer C (Eppendorf AG, Hamburg, Germany) for 15 min at 30 °C and 2000 rpm. After sample centrifugation for 5 min at 5 °C and 10,000 rpm (Centrifuge 5424 R, Eppendorf AG, Hamburg, Germany), supernatant was collected and sample pellet was resuspended in 1 mL of 70% acetone in 0.1% formic acid for a second cycle of extraction in the same conditions. Both methanolic and acetone extracts were combined, and final volume was adjusted to 2 mL with deionized water. Extracts (1 mL) were mixed with 100 µL of 0.1% FBBB reagent in distilled water and 100 µL of 5% NaOH. Sample solutions were mixed and incubated for 120 min at room temperature, avoiding light exposure. Absorbance was measured at 420 nm in a Synergy HT microplate reader (BioTek Instruments, Winooski, VT, USA). Calculations were performed using a gallic acid standard curve (0 to 225 µg/mL) (Merck, Darmstadt, Germany), and results were expressed as mg gallic acid equivalents (GAE)/100 g dw.

### 2.7. γ-Aminobutyric Acid (GABA)

For GABA extraction, 200 mg flour, pasta, or digestates was resuspended in 2 mL of 0.1 N HCl and incubated for 30 min at 5 °C and 2000 rpm in a Thermomixer C (Eppendorf, Madrid, Spain). After centrifugation for 10 min, at 5 °C and 8000× *g* (Centrifuge 5424 R, Eppendorf AG, Hamburg, Germany), supernatants were filtered using a 0.22 μm nylon syringe filter. GABA analysis was performed by reversed-phase high-performance liquid chromatography (RP-HPLC) and UV detection after pre-column derivatization with 9-fluorenylmethoxycarbonyl chloride and ortho-phthaldialdehyde reagents. Chromatographic separations were carried out in an Agilent 1200 high-performance liquid chromatograph (Agilent, Santa Clara, CA, USA) equipped with a G1314B diode array detector (DAD), and a Zorbax Eclipse Plus C_18_ stationary phase column (4.6 × 150 mm, 3 μm). The mobile phase A was composed of 10 mM Na_2_HPO_4_: 10 mM Na_2_B_4_O_7_, pH 8.2: 5 mM NaN_3_ and the mobile phase B consisted of acetonitrile: methanol: water (45:45:10, *v*/*v*/*v*). All mobile-phase solvents were HPLC-grade. Analysis was performed at 40 °C, with a flow rate of 1.5 mL/min and the following solvent gradient: 57% B in 20 min, 100% B in 20.1 min, 100% B in 23.5 min, 2% B in 23.6 min, 2% B in 25 min. The DAD detector was set to 338 nm (from 0–15 min) and 262 nm (from 15–30 min). External calibration was carried out using a standard solution of GABA (linear concentration range between 10 and 1000 pmol/μL, R^2^ ≥ 0.99). Data were expressed as mg/100 g dw.

### 2.8. Oxygen Radical Antioxidant Capacity (ORAC)

The antioxidant activity was determined in extracts (see Section 2.6) by the oxygen radical absorbance capacity (ORAC) method [11]. Briefly, reaction mixtures [30 μL of sample or the standard 6-hydroxy-2,5,7,8-tetramethylchroman-2-carboxylic acid (Trolox) in 75 mM phosphate buffer at pH 7.4, 180 μL of 70 nM fluorescein and 90 μL of 12 mM 2,2′-azobis(2-amidinopropane) dihydrochloride] were placed in black 96-well plates and relative fluorescence units were read every 2 min for 2.5 h at 485 and 520 nm for excitation and emission wavelengths, using a Synergy HT microplate reader (BioTek Instruments, Winooski, VT, USA). Data were expressed as μmol Trolox equivalents (TE)/g dw using a Trolox standard curve (concentration range from 0 to 160 μM; R^2^ ≥ 0.99).

### 2.9. Determination of Optimal Cooking Time

Optimal cooking time for each pasta type was determined following the approved 66–50 method (AACC 2000). Briefly, the minimum cooking time was determined as the cooking time at which the starchy core disappeared (qualitatively) upon squeezing between two glass plates. Then, the optimal time was determined as the minimal cooking time plus one minute. For C, PQC, and PKC, optimal times were 12, 10, and 8 min, respectively.

### 2.10. Simulated Gastrointestinal Digestion

The in vitro digestion of cooked pasta was carried out in duplicate using the SI Analytics automatic titrator (TitroLine 7000-M1, Paris, France), which recorded the volume expenditure of pH-adjusting solutions at each digestion phase. The simulated salivary (SSF), gastric (SGF), and intestinal (SIF) fluids were prepared (1.25 times concentration) and added according to the INFOGEST 2.0 method [19].

In the oral phase, 3 g of sample was dispersed in 3 mL of SSF without human salivary α-amylase. The oral bolus (6 mL) was incubated for 2 min at 37 °C. In the gastric phase, 5.1 mL of SGF containing porcine pepsin solution (EC 3.4.23.1, final concentration of 2000 U/mL, activity 3042.4 U/mg) was added. The pH was adjusted to 3.0 with 2 M HCl, and distilled water was added to reach a final volume of 12 mL. The sample was incubated for 2 h at 37 °C under constant stirring, and the pH was automatically adjusted (3.0) by the titrator using 0.3 N HCl. At the end of this stage, 6 mL of gastric digest was reserved for subsequent analyses, while in the remaining volume, the gastric digestion was stopped by adjusting the pH to 7.0 with 1 M NaOH. To initiate the intestinal phase, 4.8 mL SIF containing porcine pancreatin (EC 232.468.9, to reach a final concentration of 100 U of trypsin activity/mL, trypsin activity 5.78 U/mg) and porcine bile extract (1 mM final concentration, concentration of bile salts 18.36 mg/mmol) was added. The pH was increased to 7.0 using 1 M NaOH, and distilled water was added to adjust the final volume to 12 mL. The incubation was carried out under the same parameters as the GP, and pH was maintained at 7.0 using 0.2 N NaOH. Intestinal digestion was stopped by heat treatment at 80 °C for 10 min. The gastric and intestinal digests were freeze-dried and stored at 4 °C in vacuum-sealed plastic bags.

### 2.11. Determination of Mineral Bioaccessibility

The mineral bioaccessibility was determined by the analysis of the mineral content (see Section 2.1) from the soluble fraction of digestive supernatants (see Section 2.10). Mineral bioaccessibility was evaluated at the end of small intestinal phase and was calculated using Equation (4):

(4) $$Mineral\;bioaccessibility\;\left(\%\right)=\frac{Mineral\;content\;in\;the\;soluble\;fraction}{Total\;mineral\;content\;in\;sample}\times 100\;$$

### 2.12. Determination of GI

The GI of the cooked pasta made from optimized formulations was measured following the method of Goñi et al. [20] with modifications. Digestive supernatants (see Section 2.10) were collected at different time points (0, 5, 10, 20, 40, 60, 90, and 120 min) of the intestinal phase. The enzymatic activity was stopped by boiling tubes in a water bath for 5 min, then these were centrifuged for 5 min at 10,000× *g* and 4 °C. Afterward, 500 μL of supernatant was incubated with 100 μL of amyloglucosidase (3.3 U/mL) for 45 min at 60 °C and 1000 rpm. The glucose content in each sample was measured using the D-Glucose Assay Kit (GOPOD). Hydrolyzed starch (HS) percentage was calculated by multiplying the released glucose by 0.9 and dividing this value by the total starch content (see Section 2.1). The experimental data for starch hydrolysis were subjected to a nonlinear regression model using GraphPad Prism 4.00 software (GraphPad Software, Inc., San Diego, CA, USA). This model was integrated to calculate the area under the curve (AUC), representing the glucose concentration released over time. The hydrolysis index (HI) was obtained based on the relationship between the area under the hydrolysis curve (AUC) for each sample and AUC for the glucose standard. The estimated GI was calculated from the hydrolysis index (HI) values using Equation (5):

(5) Glycemic index (GI)=39.71+0.549×HI

### 2.13. Statistics

Preparation of pasta, digestion, and chemical analysis of samples were performed in duplicate. Results were expressed as mean ± standard deviation. Differences between the studied parameters were evaluated by a one-way analysis of variance (ANOVA). Bonferroni post hoc test was conducted to discriminate between mean values with 95% confidence intervals.

## 3. Results and Discussion

### 3.1. Nutritional Quality of SQF, SKF, and CuF Is Remarkably Higher Than Refined WF

The nutritional composition of the flours used in the present study for pasta making is detailed in Table 1. A comparative analysis of refined wheat (WF), sprouted pseudocerals (SQF and SKF), and cushuro (CuF) flours revealed distinct nutritional and phytochemical profiles (Table 1). In comparison to WF, SQF and SKF exhibited lower starch content, and higher TDF, IDF, protein, fat, ash (minerals), and PA were observed for (*p* < 0.05, Table 1), consistent with findings from previous studies [21,22,23]. In general, all analyzed minerals were present in significantly higher concentrations in SQF and SKF compared to WF, except for iron (Fe), which was found in slightly lower concentration in SQF compared to WF, in consistency with observations reported for iron content in quinoa and wheat grains in the review by Campos-Rodríguez et al. [24].

During the wheat-milling process, the removal of bran leads to a substantial loss of DF, minerals, and phenolic compounds [25]. This is in line with the lower nutritional value observed in WF compared to SQF and SKF. The increased protein, fat, and ash content observed in SQF and SKF compared to ungerminated grains is attributed to the generation and mobilization of reserves as well as the loss of total dry weight resulting from metabolic nutrient loss [10]. The reported increased in DF during the germination of quinoa and kiwicha in previous studies is explained by cell wall biosynthesis and/or the loss of total dry matter caused by the degradation of other constituents during germination [26]. Furthermore, seed germination is associated with improved mineral bioaccessibility due to activation of phytases during grain sprouting [27]. Phytases hydrolyze PA during germination, reducing its mineral-binding capacity and increasing the bioaccessibility of minerals. Our previous studies have demonstrated that the germination of quinoa and kiwicha leads to a reduction in PA content [22].

In terms of bioactive compounds, both sprouted pseudocereal flours proved to be superior sources of TSPC and GABA compared to WF (Table 1). The GABA content in cereals and pseudocereals is typically low, with maximum reported contents in the literature for wheat and quinoa not exceeding 15.50 and 66.10 mg/100 g, respectively [28]. However, germination significantly increases GABA content in pseudocereal flours, reaching up to 100–217 mg/100 g for quinoa and kiwicha, thereby enhancing their nutritional value as ingredients for developing healthier foods [11,12,22]. In the present study, the GABA content of SQF and SKF was more than 10 times higher than that of WF (Table 1). Germination activates the activity of endogenous glutamate decarboxilase enzyme, catalyzing the conversion of L-glutamic acid to GABA, thereby increasing its concentration in sprouted grains [29,30].

Similarly, the TSPC of sprouted pseudocereal flours was found to be between 2.6 (for SKF) and 10 (for SQF) times higher than the amounts found in WF (Table 1). Commercial white flour is characterized by its reduced phenolic content, a consequence of the removal of outer layers during the milling process, where phenolic compounds are concentrated [31]. Germination improves the TSPC in kiwicha and quinoa grains [22] due to de novo synthesis of phenolics or the release of bound phenolic compounds through the action of carbohydrolyses, proteases, and lipases responsible for the hydrolysis of reserve macronutrients and cell walls [29].

Higher ORAC values were observed for SQF and SKF compared to WF (Table 1). The elevated antioxidant capacity in cereal and pseudocereal flours is often correlated with a higher content of phenolic compounds [32]. However, it is important to note that the antioxidant properties of cereal and pseudocereal grains depend not only on phenolic compounds but also on other antioxidants.

The nutritional composition of CuF aligns with earlier reported values [7,8]. This microalga was enriched in protein (46.76 g/100 g dw, the highest value vs. WF, SQF and SKF; *p* ≤ 0.05, Table 1) and fiber with an IDF-to-SDF ratio of 1:3. CuF exhibited the lowest fat content among all the studied flours (*p* < 0.05, Table 1). In comparison to other flours, CuF was particularly enriched in minerals, especially essential elements such as Fe (24 mg/100 g), Mn (17.24 mg/100 g) and Ca (2161.69 mg/100 g). This nutritional profile, coupled to the low PA content, supports the hypothesis of a higher mineral bioaccessibility of CuF compared to WF, SQF, and SKF. In terms of bioactive compounds, TSPC content was higher in CuF compared to WF and SKF (*p* < 0.05, Table 1). However, the concentration of GABA and antioxidant activity in CuF were comparable to WF.

### 3.2. Modelization of the Effect of Substitution Ratio of WF with Sprouted Pseudocereals and Cushuro on PA, GABA, TSPC, and Antioxidant Activity in Pasta

**Figure 1:**
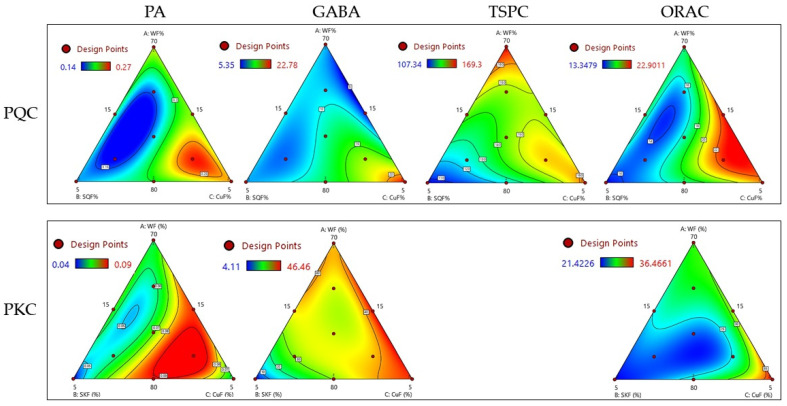
Effect of formulation of composite flour on phytic acid (PA). Total soluble phenolic compounds (TSPC) γ-aminobutyric acid (GABA); and oxygen radical absorbance capacity (ORAC) of PQC and PKC.

Modeling the impact of substituting refined wheat flour (WF) with sprouted pseudocereals and cushuro on phytic acid (PA), Gamma-Aminobutyric Acid (GABA), total soluble phenolic compounds (TSPC), and antioxidant activity in pasta involves developing a mathematical representation of the relationship between the substitution ratio and these variables. This can be achieved through statistical modeling techniques, such as regression analysis or mathematical modeling. The changes in the content of PA, GABA, TSPC, and antioxidant activity were investigated as a function of formulation of composite flour (Table S2). With the aim of understanding the effect of the independent variables, results were plotted (Figure 1). In each dependent variable, the factors that had a significant effect (*p* < 0.05) were adjusted, constituting the best mathematical model capable of predicting the behavior of the dependent variable (Table 2). The value of R^2^ for each model was >0.9 (with the exception of the regression model for TSPC in PKQ that was not used for predictive purposes). It is noteworthy that the high R^2^ values and the similarity between predictive R^2^ and adjusted R^2^ enhanced the confidence in the models’ ability to accurately predict the behavior of the dependent variables. This comprehensive approach to statistical modeling provided a robust foundation for understanding and optimizing the formulation of composite flour in relation to PA, GABA, TSPC, and antioxidant activity.

PA is acknowledged as an antinutritional factor due to its capacity to bind divalent ions, including Ca^2+^, Fe ^2+^, Mg^2+^, Mn^2+^, and Zn^2+^ [33]. Consequently, the optimization goal for composite flour was the reduction of PA content in pasta. The alterations in the PA content of raw PQC and PKC following the partial substitution of WF with sprouted pseudocereals flour and CuF are summarized in Table S2 and subjected to regression analysis to build the mathematical model described in Table 2. The regression model for PQC and PKC showed that the impact of substitution ratios is not purely linear, with significant linear, quadratic, and interaction effects of substitution ratio. For PQC, the positive coefficients (0.23A, 0.16B, 0.25C) imply that increasing the substitution ratios of WF with sprouted quinoa and cushuro generally leads to an increase in PA. The negative interaction term (−0.08AC) indicated that the combined effect of wheat flour (A) and cushuro (C) may have a negative impact on PA when both are increased simultaneously. For PKC, the negative quadratic term (−2.00A^2^BC) suggested that the relationship involving the squared term of wheat flour may introduce nonlinearity, potentially impacting PA negatively. The PA content in PQC and PKC varied within the ranges of 0.14–0.27 and 0.04–0.09 g/100 g dw (Table S2), respectively. Notably, the PA content exhibited a consistent decrease as the substitution ratios of sprouted pseudocereals (SQF and SKF) decreased and WF and CuF increased in pasta formulation (Figure 1). Specifically, the lowest PA content was generally observed when the SQF/SKF ratio ranged between 5% and 13% and the CuF ratio was between 12 and 15% (Table S2; Figure 1). This outcome was attributed to the initially higher PA content in SQF and SKF (1.21 and 0.58 g/100 g dw, respectively; Table 1) in comparison to the lower PA content in CuF and WF (0.05 and 0.17 g/100 g dw; Table 1).

GABA functions as an inhibitory neurotransmitter within the central nervous system and boasts a wide array of promising bioactivities, including antihypertensive, immunomodulatory, antidiabetic, and antitumoral properties, among others [28]. Consequently, the present study sought to develop functional pasta enriched with GABA, emphasizing the optimization of the composite flour formulation to maximize GABA concentration. The GABA concentration in pasta was notably influenced by the substitution ratio of WF with SQF/SKF and CuF (Figure 1, Table 2 and Table S2). The regression models outlining the influence of the WF (A), SQF/SKF (B), and CuF (C) ratios in the composite flour on the GABA content is shown in Table 2. Increasing the substitution ratio of WF, SQF/SKF, and CuF positively contributes to GABA content. There are negative interaction effects, indicating that the simultaneous increase in certain pairs of substitution ratios (e.g., WF and CuF) leads to a decrease in GABA content. The quadratic term suggested that the relationship between the three variables is not strictly linear but involves more complex, potentially nonlinear dynamics. The GABA content was observed to vary within the ranges of 8.13–22.78 mg/100 g and 4.11–46.46 mg/100 g dw for PQC and PKC, respectively (Table S2). Notably, this ranges in certain instances, exceeded the GABA content found in the C pasta (14.75 mg/100 dw). Furthermore, the investigation into the effect of substitution ratios on GABA content revealed that the highest concentrations of GABA were achieved when the ratios of SQF/SKF were at 15% and CuF was at 5% (Table S2; Figure S1). This observation led to the conclusion that in summary, optimizing the formulation by incorporating high levels of sprouted pseudocereals and minimizing the inclusion of cushuro proved effective in enhancing the GABA content in the wheat-based pasta, potentially offering additional nutritional benefits.

The composition of pasta, specifically the ratios of WF, SQF, and CuF flours, had a multifaceted impact on the TSPC. In this model, the individual variables (WF, SQF and CuF) had substantial influences on TSPC in PQC, but their combined effect, especially in a quadratic relationship, appears to be the most influential in this model (Table 2). The content of TSPC in PQC and PKC varied between 107.34–169.03 and 143.12–227.06 mg GAE/100 g DW, respectively (Table S2). This range suggested an increase in the TSPC content compared to the control pasta (121.03 mg GAE/100 g DW, Table 3). In Figure 1, the impact of different substitution ratios is visualized, revealing a more pronounced effect of the SQF/SKF ration compared to the CuF. The highest TSPC content was achieved at high ratios of both SQF and CuF, specifically when these ingredients were present in the composite flour at ratios ranging between 10% and 15% (Table S2; Figure 1). This indicates that an optimal combination of SQF/SKF and CuF ratios contributed to maximizing the TSPC in the pasta (Table S2; Figure 1).

Models indicates that the composition of the pasta, specifically the ratios of WF, SQF/SKF, and CuF, has a complex and potentially nonlinear impact on antioxidant activity in PQC and PKC (Table 2). The interactions and quadratic terms highlight the importance of considering not only the individual flours but also their combined effects on the antioxidant activity in the pasta. ORAC varied between 13.35 and 22.90 µmol TE/g dw in PQC and between 21.42 and 36.47 µmol TE/g dw in PKC (Table S2), values higher than those observed for control pasta (2.42 µmol TE/g dw). Figure 1 and Table S2 shows higher antioxidant activity when SQF/SKF and CuF ratios were between 13–15% and 5–10% in the composite flour, respectively.

All results combined showed that an adequate combination of sprouted pseudocereals and CuF ratios in composite flour significantly contributes to maintain a compromise to reduce PA levels and to enhance GABA and TSPC concentration and antioxidant activity in wheat-based pasta.

### 3.3. Optimal Supplementation Ratio of WF with SQF/SKF and CuF Blends Improved the Nutrional Quality and Functional Value of Wheat-Based Pasta

To find the optimal formulation of composite flour for the enhancement of GABA, TSPC and antioxidant activity while minimizing PA concentration, a multiple response optimization was performed. This procedure helped in determining the combination of WF, SQF/SKF, and CuF ratios, giving equal importance to all responses (Table 3). The optimum value reached for desirability was 0.59 for PQC. The optimal formulation provided in the multiple response optimization for PQC was 79% WF, 13% SQF, and 8% CuF, for which the predicted values for PA, GABA, TSPC, and ORAC were 0.25 g/100 g dw, 20.28 mg/100 mg dw, 160.08 mg GAE/100 g dw, and 22.03 μmol TE/g dw, respectively.

Regarding PKC, the optimal formulation was 70% WF, 15% SKF, and 15% CuF (optimum desirability value = 0.81), for which the predicted values reached 0.06 g/100 g dw for PA, 37.91 mg/100 mg dw for GABA, 215.12 mg GAE/100 g dw for TSPC, and 29.64 μmol TE/g dw for ORAC.

The experimental values for validating each response were compared to the predictions made by the mathematical model. The tests were conducted in triplicate, and the corresponding values are also detailed in Table 3. The validation of the mathematical models was confirmed, as the relative deviation was consistently below 5% for the majority of response variables.

The chemical composition of the pasta samples prepared with optimal recipes is summarized in Table 4. The partial substitution of WF with SQF/SKF and CuF at optimal supplementation ratios (13% SQF and 8% CuF for oPQC; and 15% SKF and 15% CuF for oPKC) resulted in a decrease in starch content and an increase in fat, protein, TDF, IDF, SDF (for oPKC), and ash.

Similarly, the mineral content (K, Na, Fe, Zn, Mg, Mn, and Ca), TSPC, GABA, and antioxidant activity values were significantly higher in oPQC and oPKC compared to control pasta (C, Table 4). This can be attributed to the nutritional profiles of SQF/SKF and CuF compared to WF (Table 1). Similar findings have been reported in pasta with 10–30% supplementation of SQF [34], where the increase was attributed to the high levels of protein and minerals in germinated quinoa. Moreover, the incorporation of 2–15% microalgae (Spirulina) into semolina-based pasta led to increased protein, lipid, Fe, and Ca contents up to 77.47, 37.60, 296.99, and 57.27% (dw), respectively [35]. Pasta enriched with Spirulina, at different levels of addition, also showed higher phenolic content, flavonoids, and antioxidant activity compared to the control, supporting our results.

Comparing oPQC and oPKC, the latter pasta prototype exhibited superior nutritional value with significantly lower starch and higher levels of TDF, IDF, SDF, ash, K, Mg, Ca, GABA, TSPC, and ORAC. This difference is attributed to the higher supplementation ratio of WF with SKF (15%) and CuF (15%) in oPKC as compared to oPQC (13% SQF and 8% CuF).

In summary, the optimized pasta formulations, enriched with sprouted pseudocereal flours and cushuro, offer promising practical implications for consumer health. The substantial increase in essential nutrients such as dietary fiber, protein, minerals (K, Na, Fe, Zn, Mg, Mn, Ca), and bioactive compounds (TSPC, GABA) in the optimized pastas suggests a significant leap in nutritional quality. These components play crucial roles in supporting various aspects of human health, including immune function, bone health, and antioxidant defense.

### 3.4. Effect of Cooking on Nutritional Composition and Bioactive Value of Optimized Pasta Types

After cooking, the starch content did not undergo significant changes for all pasta types (*p* > 0.05, Table 4). Fat content decreased in oPQC and oPKC (*p* < 0.05, Table 4), while in the control pasta (C, 100% WF), it remained without a significant variation (*p* ≥ 0.05, Table 4). Compared to raw pasta, the protein content slightly increased for all cooked pasta types (*p* < 0.05, Table 4), indicating that protein did not leach into the cooking water. This is mainly attributed to the migration of soluble components into the water during cooking [36].

oPQC and oPKC can be labeled as a protein source since, according to Regulation (EC) No. 1924/2006, its protein content contributes more that 12% of the energy value of the final product. Considering a serving size of dry pasta as 100 g dw, a serving of oPQC and oPKC, upon cooking, provides approximately 17 g of protein, which is 30.3–37.7% of the recommended protein intake for adults (~56 g/day for men and ~45 g/day for women).

A variation in the cooking effect on fiber fraction was observed among the different pasta types. In the case of C (100%WF), TDF decreased after cooking due to SDF leaching, as reported in previous studies [37]. While a slight reduction in SDF was also noted for oPQC and oPKC (%), TDF and IDF increased significantly after cooking (*p* < 0.05). These findings align with the study by Sobota and Zarzycki [37], suggesting that cooking impacts TDF, IDF, and SDF of pasta products, and that impact is dependent on pasta type and cooking time. The consumption of a portion of 100 g dw of cooked oPQC and oPKC pasta supplies around 17% of TDF covering 57–68% of the daily recommendation for DF intake (25–30 g/d).

During the cooking process, the ash content experienced an increase in the C pasta, while values remained stable for optimized pasta types (oPQC and oPKC). Specifically, the K and Na content of all three pasta types significantly decreased upon cooking. This is attributed to the absence of interactions with chelators, allowing K and Na ions to leach more easily compared to other minerals, as discussed in earlier reviews [38]. In contrast, the Fe content did not significantly decrease after cooking for any of the considered pasta types in accordance to previous studies showing that Fe content remained unchanged after lentil pasta cooking [39]. In pulses, cereals, and pseudocereals, nonferritin Fe preferentially binds with phytates, minimizing leaching during cooking [40,41]. Conversely, the Mg, Mn, and Zn content of the optimized pasta types significantly increased upon cooking, while no significant variation was found in these minerals for the C pasta. The higher PA content of optimized pasta types could favor interactions of Mg, Mn, and Zn with this chelator; therefore, the leaching of these minerals is limited during the cooking of PQC and PKC. No significant decrease in Ca content was observed upon pasta cooking for C and PQC; in contrast, a significant reduction in Ca content was observed upon cooking in PKC, similarly to the cooking of Bambara groundnuts and common beans [40,42]. Overall, there was a general increase in PA content upon cooking for all pasta types.

Regarding bioactive compounds, there was a significant decrease in GABA for all pasta types, attributed to its leaching into water during the cooking process. This aligns with findings in germinated brown rice, where the GABA content significantly dropped from 15.0 mg/100 g to 8.5 and 8.4 mg/100 g after boiling water and pressure cooking, respectively [43]. Despite GABA’s generally good thermal stability, our results are consistent with the understanding that heat treatment above 105 °C can lead to a reduction in GABA content in pasta owing to thermal decomposition [44].

Similarly, TSPC content witnessed reductions of 28%, 8%, and 18% during the cooking process for C, oPQC, and oPKC, respectively (*p* < 0.05, Table 4). This trend was mirrored in the antioxidant activity, which decreased by 27%, 45%, and 46% after cooking for C, oPQC, and oPKC, respectively. These outcomes align well with the existing literature. Conti et al. [45] noted that phenolic content and antioxidant activity in cooked pasta enriched with carrot and olive leaf were lower than in uncooked pasta, attributed to the leaching of polyphenols into the cooking water. In a similar vein, Suo et al. [46] reported percentages losses of 24.68%, 33.49%, and 38.85% for TSPC in control, ancient, and pigmented wheat pastas, respectively, while the corresponding percentages of loss of antioxidant activity were 59.23%, 59.68%, and 2.45%. As previously mentioned, the presence of oxygen, water, and heat treatment during pasta processing induces the oxidative degradation of antioxidants [47].

### 3.5. Optimal WF Supplementation with SQF and CuF Reduces Starch Digestion and GI in Pasta

**Figure 2:**
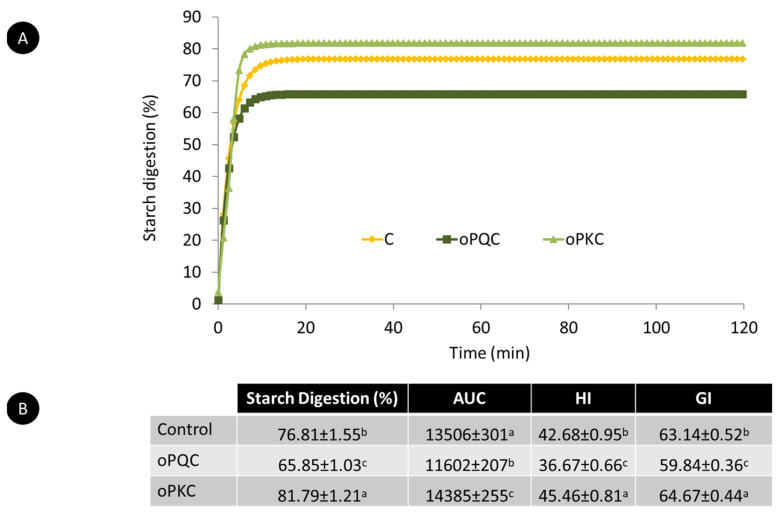
(**A**) In vitro amylolysis kinetic of C, oPQC, and oPKC cooked to its optimal cooking time. (**B**) Percentage of starch hydrolysed upon digestion, area under the curve, hydrolysis index and glycemic index of C, oPQC, and oPKC. Data are means ± standard deviation (*n* = 2). Different letters denote statistical differences among samples (ANOVA, Bonferroni post hoc test, *p* < 0.05). Abbreviations: AUC, area under the curve; C, control pasta formulated with 100% refined wheat flour; GI, glycemic index; HI, hydrolysis index; oPKC, optimized wheat-based pasta supplemented with 13% sprouted kiwicha and 8% cushuro flour; oPQC, optimized wheat-based pasta supplemented with 15% sprouted quinoa and 15% cushuro flour.

To our knowledge, the impact of supplementing WF with sprouted pseudocereals and CuF on starch digestibility in wheat-based pasta has not been previously explored. The kinetics of starch hydrolysis during in vitro small intestinal digestion for the three pasta types are presented in Figure 2. Initially, all pasta types exhibited a phase of increasing amylolysis, reaching a plateau with short digestion times (Figure 2A). Notably, oPKC demonstrated a more pronounced amylolysis (81.79%) after 120 min of in vitro small intestinal digestion, surpassing both C (76.8%) and oPQC (65.85%). This is corroborated by significant differences in the area under the curve (AUC), hydrolysis index, and glycemic index (GI) as illustrated in Figure 2B. The GI of oPQC was notably lower (59.84) compared to C (63.14) and PKC (64.67). These findings suggest that incorporating 13% SQF and 8% CuF into wheat-based pasta could effectively diminish the rate and extent of starch hydrolysis in vivo, showcasing their potential to delay starch hydrolysis in such applications. Similarly, a study by Xu et al. [48] observed a decrease in starch digestibility in wheat/quinoa bread compared to control bread (100% WF).

Amylolysis was lower in the oPQC as compared to the oPKC, which could be attributed to the differences in the morphological and physicochemical properties of quinoa and kiwicha starches. Quinoa starch has lower swelling power and solubility (in a temperature range from 40 to 90 °C) than kiwicha starches [49]. Additionally, quinoa starch demonstrates higher organization of crystalline structures, with amylopectin having a greater molecular weight and a more compact structure within quinoa starch granules than in kiwicha starch [49]. These differences in starch characteristics could, in part, account for the reduced susceptibility of oPQC starch to enzymatic hydrolysis by pancreatic α-amylases compared to oPKC.

Furthermore, the slightly higher wheat flour supplementation ratio in oPKC (15% SKF and 15% CuF) compared to oPQC (13% SQF and 8% CuF) may have led to a minor increase in starch hydrolysis during pasta digestion due to a more significant dilution effect on the gluten matrix. In pasta, starch granules are embedded within the continuous gluten network, limiting starch swelling and reducing the accessibility of starch to α-amylase [50]. The elevated fiber and protein (nongluten) content in oPKC might disrupt the continuity of the gluten matrix, making starch granules more vulnerable to enzymatic degradation and increasing starch digestion, as previously observed with the addition of native and sprouted quinoa flour in pasta [34,49,51]. Furthermore, quinoa starch exhibits a more organized crystalline structure, with higher-molecular-weight amylopectin and a more compact structure within its granules compared to kiwicha [49]. These differences in starch characteristics could partially explain the lower susceptibility of oPQC starch to enzymatic hydrolysis by pancreatic α-amylases than oPKC.

Additionally, the higher wheat flour supplementation ratio in oPKC (15% SKF and 15% CuF) compared to oPQC (13% SQF and 8% CuF) may have slightly increased starch hydrolysis during pasta digestion due to a more pronounced dilution effect on the gluten matrix. In pasta, starch granules are embedded within the continuous gluten network, limiting starch swelling and reducing accessibility to α-amylase [50]. The elevated fiber and protein (nongluten) content in oPKC could disrupt the continuity of the gluten matrix, making starch granules more vulnerable to enzymatic degradation, thus increasing starch digestion. This aligns with previous findings on the impact of adding native and sprouted quinoa flour to pasta [34,51].

The observed lower GI of the optimized pasta formulations, especially oPQC, indicates potential benefits for individuals managing blood sugar levels. This characteristic could be particularly advantageous for those with diabetes or individuals seeking to control their blood sugar responses to food intake.

### 3.6. Optimal WF Supplementation with SQF and CuF Reduces Starch Digestion and GI in Pasta

Table 5 presents the alterations in GABA, TSPC, and antioxidant activity during simulated gastrointestinal digestion of cooked pasta samples. GABA bioaccessibility in gastric and intestinal digestates from cooked pasta samples ranged from 81% to 85% and 74% to 100%, respectively. These values are consistent with reported GABA bioaccessibility in gastric and intestinal digestates of cookies and extrudates supplemented with sprouted kiwicha and quinoa flours [23]. As anticipated, gastric and intestinal digestates from cooked oPQC and oPKC pasta types exhibited higher bioaccessible GABA content compared to the control (C) (*p* < 0.05), attributable to their higher initial GABA content (Table 4). Furthermore, bioaccessible GABA levels were significantly elevated in oPKC compared to oPQC (*p* < 0.05).

The bioaccessible concentration of TSPC showed a decrease at the conclusion of the gastric phase for all cooked pasta types (*p* < 0.05, Table 5). As the digestion process advanced, TSPC levels increased at the end of the intestinal phase, reaching levels equal (for C) or higher (for oPQC and oPKC) than those observed for undigested samples. Both oPQC and oPKC pasta exhibited significantly higher (*p* < 0.05) bioaccessible TSPC content compared to C pasta after in vitro digestion. These findings align with those reported by Wang et al. [52], who observed a substantial increase in TSPC values in fortified pasta samples with juice, puree, and pomace from red cabbage and spinach. In cereals, phenolics can form conjugates with sugars, cell wall polysaccharides, or amines [53]. In vitro digestion has the potential to release these conjugated phenolics as starch and proteins, to which these phenols are bonded, undergoing hydrolysis [54]. In this study, the TSPC value of intestinal digests did not significantly increase in C pasta, which could be explained by its low content of bound phenolics.

Throughout the gastrointestinal transit of all cooked pasta types, antioxidant activity gradually increased, reaching the highest values at the end of the intestinal phase. The antioxidant activity of gastric and intestinal digestates from oPQC and oPKC was higher compared to C (*p* < 0.05, Table 5), suggesting that the inclusion of sprouted pseudocereals and cushuro flour can enhance the radical scavenging activity of pasta samples. This aligns with prior studies that focused on incorporating sprouted kiwicha, quinoa, and cañihua flours into cookies and extrudates [21,22]. The antioxidant activity of pasta samples is contingent on factors such as phenolic content, vitamin C, vitamin E and carotenoids. Similarly, Wang et al. [52] observed that both digested control and spinach-enriched pasta exhibited elevated antioxidant activity compared to their cooked counterparts.

Deficiencies in essential minerals such as Ca and Fe pose significant public health challenges globally [55]. Micronutrient status studies reveal high rates of anemia, affecting 33–40% of children and women, with 37% experiencing multiple micronutrient deficiencies. Addressing mineral deficiencies is crucial, and investigations into mineral bioaccessibility play a vital role in this context. Table 5 presents the bioaccessibility of Ca and Fe in cooked pasta samples—C, oPQC, and oPKC. Consistent with prior research on legume, cereal, and pseudocereal-based pasta [39], mineral bioaccessibility did not reach 100%, suggesting notable mineral chelation.

Ca bioaccessibility varied significantly among the different pasta samples, with values ranging from 24% to 66%. Notably, C pasta exhibited the highest Ca bioaccessibility at 66%, surpassing cooked oPQC (40%) and oPKC (24%). This indicates a substantial impact of incorporating pseudocereal and cushuro flour into pasta on Ca bioaccessibility. Intriguingly, despite having a lower Ca content (Table 4), cooked C pasta demonstrated higher bioaccessibility, possibly due to increased Ca release during digestion. C pasta, composed mainly of starchy endosperm, provided a simpler food matrix that is easily digested by enzymes, facilitating Ca release. In contrast, the SQF/SKF and CuF added to the WF altered the food matrix composition, enriching it with fiber, proteins, polyphenols, and phytic acid (PA), known Ca-chelating agents [41]. The dietary fiber has an adverse effect on Ca bioavailability due to its binding capacity with PA. The higher content in fiber observed for oPQC and oPKC may explain its lower Ca bioaccessibility compared to C pasta. However, at the end of intestinal digestion, the bioaccessible Ca concentration was 5.6 and 3.5 times higher in oPQC and oPKC pasta, respectively, compared to C pasta. A consumption of a portion of 100 g of cooked C, oPQC, and oPKC pasta (dry basis) could offer around 2.4%, 12.5%, and 8.34%, respectively, of the average daily requirement for Ca (700 mg/day) (EFSA NDA Panel, 2010).

The bioaccessibility of iron (Fe) was generally low across all pasta samples, ranging from 7.3% to 8.2%. Slightly lower bioaccessible Fe concentrations were observed in oPQC and oPKC compared to C pasta. This difference could be attributed to the higher PA and phenolic compounds content in the optimized pasta types. Phytic acid, lower inositol phosphates, and iron-binding phenolics are inhibitors that reduce Fe bioavailability [41]. The contribution to the average daily requirement for Fe (6 mg/day) of a portion of 100 g of cooked C, oPQC and oPKC (dry basis) would deliver considerable bioaccessible amounts of Fe (up to 45.5, 46%, and 42%, respectively).

The insights gained from the in vitro digestion study highlight the increased bioaccessibility of key nutrients and bioactive compounds. This knowledge can guide the development of food products that optimize nutrient absorption during digestion, aligning with the growing emphasis on bioavailability in functional foods.

## 4. Conclusions

In this study, various pasta prototypes were formulated by supplementing white wheat flour with sprouted pseudocereals (quinoa and kiwicha) and cushuro flour. The enrichment of pasta with a combination of sprouted quinoa (13%) and cushuro (8%) and sprouted kiwicha (15%) and cushuro (15%) resulted in significant higher levels of protein, fat, soluble dietary fiber, and ash content compared to control wheat pasta. The pasta prototype enriched with sprouted pseudocereals and low levels of cushuro (oPKC) demonstrated the highest concentrations of GABA, suggesting its potential as a functional food for promoting health. The optimized pasta types (oPQC and oPKC) exhibited higher TSPC content compared to the control pasta (C), indicating increased levels of bioactive compounds with potential health benefits. While the optimized pasta types showed elevated mineral content, particularly in K, Na, Fe, Zn, Mg, Mn, and Ca, the bioaccessibility of Ca and Fe was influenced by higher phytic acid content, emphasizing the importance of considering both mineral content and bioavailability. Enhanced antioxidant activity was observed in the optimized pasta types (oPQC and oPKC) during simulated gastrointestinal digestion, reinforcing their potential health-promoting properties. Starch hydrolysis kinetics revealed differences in the rate and extent of starch hydrolysis, with oPKC exhibiting higher amylolysis compared to oPQC. This suggests the potential influence of flour composition on starch digestibility. Considering the comprehensive evaluation of GABA concentration, TSPC, mineral content, antioxidant activity, and starch digestibility, oPKC emerges as a promising pasta prototype, particularly for those prioritizing GABA enrichment and elevated TSPC while being mindful of mineral bioaccessibility. However, further studies, including sensory evaluations, are recommended to assess the acceptability and palatability of these optimized pasta prototypes.

This study opens avenues for further research on the incorporation of sprouted pseudocereals and unique microbial sources like cushuro in various food products. Exploring their application in other staple foods could lead to diversified options for consumers seeking enhanced nutritional content. As these formulations differ significantly from traditional pasta, conducting consumer acceptance studies would be beneficial. Understanding consumer preferences, sensory perceptions, and willingness to adopt these innovations will be pivotal for successful market integration.

The practical implications of our research extend beyond the laboratory, offering tangible benefits for consumers and inspiring innovation in the food industry. The optimized pasta formulations present an exciting opportunity to bridge the gap between nutrition and taste, ultimately contributing to the promotion of healthier dietary choices and the evolution of the food market towards more functional and nutritious options.

## Figures and Tables

**Table 1:** Chemical composition of flours used in pasta making on a dry basis.

	Units	WF	SQF	SKF	CuF
Starch	g/100 g	70.81 ± 0.69 ^a^	55.84± 0.52 ^b^	44.69 ± 0.04 ^c^	0.16 ± 0.03 ^d^
TDF	g/100 g	8.50 ± 0.78 ^c^	18.84 ± 1.20 ^b^	23.06 ± 0.67 ^a^	19.77 ± 0.57 ^b^
IDF	g/100 g	0.73 ± 0.26 ^d^	12.34 ± 1.06 ^b^	16.18 ± 0.60 ^a^	5.53 ± 0.26 ^c^
SDF	g/100 g	7.77 ± 0.53 ^b^	6.14 ± 0.14 ^d^	6.87 ± 0.07 ^c^	14.23 ± 0.33 ^a^
Protein	g/100 g	12.44 ± 0.02 ^d^	23.36 ± 2.38 ^b^	13.87 ± 0.03 ^c^	46.76 ± 4.80 ^a^
Fat	g/100 g	0.90 ± 0.09 ^b^	6.55 ± 0.11 ^a^	6.77 ± 0.06 ^a^	0.68 ± 0.01 ^c^
Ash	g/100 g	0.52 ± 0.07 ^d^	3.66 ± 0.11 ^b^	2.09 ± 0.02 ^c^	6.17 ± 1.78 ^a^
K	mg/100 g	152.37 ± 2.58 ^a^	523.59 ± 9.20 ^b^	653.75 ± 13.63 ^a^	112.39 ± 1.66 ^d^
Na	mg/100 g	1.26 ± 0.39 ^c^	14.71 ± 0.34 ^b^	8.03 ± 0.66 ^c^	453.42 ± 2.65 ^a^
Fe	mg/100 g	5.59 ± 0.41 ^c^	4.45 ± 0.20 ^d^	8.36 ± 0.35 ^b^	24.68 ± 0.67 ^a^
Zn	mg/100 g	1.26 ± 0.03 ^c^	4.54 ± 0.07 ^b^	5.99 ± 0.13 ^a^	4.78 ± 0.17 ^b^
Mg	mg/100 g	38.09 ± 2.88 ^d^	153.27 ± 9.87 ^c^	279.42 ± 16.39 ^a^	215.36 ± 1.21 ^b^
Mn	mg/100 g	1.03 ± 0.04 ^d^	1.79 ± 0.05 ^c^	2.15 ± 0.03 ^b^	17.24 ± 0.78 ^a^
Ca	mg/100 g	17.09 ± 1.01 ^d^	129.39 ± 3.22 ^c^	206.14 ± 2.50 ^b^	2161.69 ± 64.86 ^a^
PA	g/100 g	0.17 ± 0.00 ^c^	0.58 ± 0.01 ^b^	1.21 ± 0.07 ^a^	0.05 ± 0.00 ^d^
GABA	mg/100 g	0.38 ± 0.00 ^b^	5.15 ± 0.43 ^a^	5.03 ± 0.02 ^a^	0.42 ± 0.01 ^b^
TSPC	mg GAE/100 g	55.40 ± 5.56 ^d^	525.50 ± 38.14 ^a^	144.72 ± 2.09 ^c^	306.48 ± 22.91 ^b^
ORAC	μmol TE/g	17.40 ± 1.94 ^c^	45.30 ± 3.96 ^a^	35.44 ± 4.55 ^b^	15.46 ± 1.41 ^c^

Data are means ± standard deviation (*n* = 2). Statistical differences among samples are indicated by distinct letters within a row (ANOVA, Bonferroni post hoc test, *p* < 0.05). Abbreviations: CuF: cushuro flour; GABA, γ-aminobutyric acid; GAE, gallic acid equivalents; nd: not detected; IDF: insoluble dietary fiber; ORAC, oxygen radical absorbance capacity; PA, phytic acid; SDF: soluble dietary fiber; SKF, sprouted kiwicha flour; SQF, sprouted quinoa flour; TDF: total dietary fiber; TE: Trolox equivalents; TSPC, total soluble phenolic compounds; WF: wheat flour.

**Table 2:** Predictive regression models for PA, GABA, TSPC and antioxidant activity (as determined by ORAC assay) of pasta according to the independent variables [WF (A), SQF/SKF (B) and CuF (C) ratios in the composite flour].

Pasta Type	Dependent Variables	Mathematical Models	*p*-Value	R^2^ (pred)	R^2^ (adj)
PQC	PA	y = 0.23A + 0.16B + 0.25C − 0.08AC + 4.94A^2^BC − 3.84AB^2^C + 5.88ABC^2^	0.000	0.997	0.99
	GABA	y = 8.76A + 8.22B + 22.87C − 40.42AC − 11.86BC + 365.14ABC^2^	0.014	0.98	0.96
	TSPC	y = 169.53A + 107.57B + 163.41C −77.58AC − 49.03BC + 1320.92ABC^2^	0.021	0.99	0.98
	ORAC	y = 16.66A + 13.31B + 21.60C + 8.82AB + 14.51AC − 367.85A^2^BC − 124.45AB^2^C + 248.29ABC^2^	0.001	0.99	0.98
PKQ	PA	y = 0.06A + 0.04B + 0.06C + 0.07AB + 0.11AC + 0.16BC − 2.00A^2^BC	0.013	0.94	0.83
	GABA	y = 37.91A + 4.15B + 44.63C + 62.26AB + 21.41AC + 24.57BC − 772.47A^2^BC + 584.84AB^2^C − 382.48ABC^2^	0.000	0.99	0.99
	TSPC	y = 215.12A + 174.92B + 160.65C	0.022	0.50	0.41
	ORAC	y = 29.69A + 21.33B + 36.38C − 10.25BC − 482.142ABC^2^	0.026	0.97	0.95

Abbreviations: GABA, γ-aminobutyric acid; ORAC, oxygen radical absorbance capacity; PA, phytic acid; PKC, wheat-based pasta supplemented with sprouted kiwicha and cushuro flour; PQC, wheat-based pasta supplemented with sprouted quinoa and cushuro flour; TSPC, total soluble phenolic compounds.

**Table 3:** Composition of flour blends and predicted values for PA, GABA, TSPC, and ORAC in pasta at optimum desirability value (D).

Pasta Type	Response Variables	Criteria	Importance Level	Optimum Desirability (D)	Optimal Formulation	Predicted Values	Experimental Values
PQC	PA	minimize	5	0.593	79.5% WF 13% SQF 8% CuF	0.25	0.27 ± 0.01
	GABA	maximize	5			20.28	17.70 ± 0.19
	TSPC	maximize	5			160.08	158.94 ± 7.98
	ORAC	maximize	5			22.03	22.10 ± 0.73
PKC	PA	minimize	5	0.693	70% WF 15% SKF 15% CuF	0.06	0.06 ± 0.01
	GABA	maximize	5			37.91	37.87 ± 0.65
	TSPC	maximize	5			215.12	227.06 ± 11.53
	ORAC	maximize	5			29.64	29.79 ± 2.87

Abbreviations: CI: confidence interval; CuF: cushuro flour; GABA, γ-aminobutyric acid; ORAC, oxygen radical absorbance capacity; PA, phytic acid; PKC, wheat-based pasta supplemented with sprouted kiwicha and cushuro flour; PQC, wheat-based pasta supplemented with sprouted quinoa and cushuro flour; SKF, sprouted kiwicha flour; SQF, sprouted quinoa flour; TSPC, total soluble phenolic compounds; WF: refined wheat flour.

**Table 4:** Chemical composition of raw and cooked control (C) and optimized wheat-based pastas (oPQC and oPKC) on a dry basis.

Parameter	Units	RAW			COOKED		
		C	oPQC	oPKC	C	oPQC	oPKC
Starch	g/100 g	62.95 ± 2.03 ^ab^	50.88 ± 3.34 ^bc^	42.94 ± 0.18 ^c^	68.57 ± 7.68 ^a^	59.40 ± 1.54 ^bc^	44.73 ± 3.54 ^c^
Fat	g/100 g	1.63 ± 0.03 ^cd^	2.53 ± 0.02 ^a^	2.19 ± 0.07 ^ab^	1.99 ± 0.16 ^bc^	1.87 ± 0.14 ^bcd^	1.38 ± 0.16 ^e^
Protein	g/100 g	12.88 ± 0.01 ^d^	14.90 ± 0.09 ^b^	14.97 ± 0.10 ^b^	14.08 ± 0.32 ^c^	17.04 ± 0.11 ^a^	16.92 ± 0.08 ^a^
TDF	g/100 g	5.50 ± 0.56 ^d^	8.16 ± 0.27 ^c^	19.89 ± 0.12 ^a^	1.95 ± 0.27 ^e^	12.58 ± 1.10 ^b^	19.23 ± 0.32 ^a^
IDF	g/100 g	2.33 ± 0.21 ^c^	5.25 ± 0.12 ^b^	10.37 ± 0.20 ^a^	1.40 ± 0.20 ^c^	10.95 ± 0.81 ^a^	11.44 ± 0.74 ^a^
SDF	g/100 g	3.17 ± 0.35 ^c^	2.91 ± 0.15 ^cd^	9.52 ± 0.31 ^a^	0.55 ± 0.08 ^e^	1.63 ± 0.29 ^de^	7.79 ± 0.41 ^b^
Ash	g/100 g	0.62 ± 0.10 ^c^	1.40 ± 0.10 ^b^	1.69 ± 0.07 ^a^	1.36 ± 0.01 ^b^	1.29 ± 0.08 ^b^	1.67 ± 0.11 ^a^
K	mg/100 g	129.34 ± 5.91 ^b^	205.62 ± 7.48 ^a^	204.17 ± 9.64 ^a^	45.46 ± 1.71 ^d^	101.8 ±2.89 ^c^	106.76 ± 4.09 ^c^
Na	mg/100 g	22.61 ± 0.23 ^b^	40.75 ± 2.30 ^a^	42.57 ± 0.41 ^a^	11.46 ± 1.94 ^c^	23.41 ±3.99 ^b^	14.90 ± 0.51 ^c^
Fe	mg/100 g	22.83 ± 0.51 ^c^	28.32 ± 0.86 ^ab^	29.44 ± 0.93 ^ab^	25.95 ± 1.28 ^bc^	32.12 ± 1.31 ^a^	32.04 ± 6.18 ^a^
Mg	mg/100 g	35.48 ± 0.95 ^d^	80.57 ± 1.32 ^c^	90.71 ± 1.84 ^b^	38.35 ± 0.97 ^d^	86.97 ± 2.39 ^b^	101.44 ± 0.73 ^a^
Mn	mg/100 g	0.69 ± 0.01 ^e^	11.78 ± 0.42 ^b^	8.00 ± 0.33 ^d^	0.80 ± 0.03 ^e^	13.77 ± 0.23 ^a^	9.35 ± 0.20 ^c^
Zn	mg/100 g	1.08 ± 0.35 ^e^	4.22 ± 0.13 ^b^	1.82 ± 0.03 ^d^	1.20 ± 0.05 ^d^	5.14 ± 0.14 ^a^	2.18 ± 0.04 ^c^
Ca	mg/100 g	31.24 ± 1.57 ^c^	215.62 ± 4.72 ^b^	383.07 ± 28.8 ^a^	25.17 ± 0.87 ^c^	241.13 ± 32.14 ^b^	231.90 ± 20.11 ^b^
PA	g/100 g	0.02 ± 0.00 ^d^	0.09 ± 0.01 ^c^	0.06 ± 0.01 ^cd^	0.08 ± 0.01 ^c^	0.17 ± 0.02 ^b^	0.23 ± 0.00 ^a^
GABA	mg/100 g	1.48 ± 0.04 ^d^	1.77 ± 0.02 ^c^	3.79 ± 0.06 ^a^	0.49± 0.03 ^f^	0.86± 0.02 ^e^	2.21± 0.04 ^b^
TSPC	mg GAE/100 g	121.03 ± 6.12 ^e^	158.94 ± 7.98 ^c^	227.06 ± 11.53 ^a^	87.37 ± 6.04 ^f^	146.46 ± 2.38 ^d^	186.46 ± 10.75 ^b^
ORAC	µmol TE/g	3.42 ± 0.18 ^e^	22.10 ± 0.73 ^b^	29.79 ± 2.87 ^a^	2.48 ±0.43 ^f^	12.92 ± 0.34 ^d^	15.99 ± 2.90 ^c^

Data are means ± standard deviation (*n* = 2). Statistical differences among samples are indicated by distinct letters within a row (ANOVA, Bonferroni post hoc test, *p* < 0.05). Abbreviations: C, control pasta formulated with 100% refined wheat flour; dw, dry weight; GABA, γ-aminobutyric acid; GAE, gallic acid equivalents; IDF: insoluble dietary fiber; ORAC, oxygen radical absorbance capacity; PA, phytic acid; oPKC, optimized wheat-based pasta supplemented with 13% sprouted kiwicha and 8% cushuro flour; oPQC, optimized wheat-based pasta supplemented with 15% sprouted quinoa and 15% cushuro flour; SDF: soluble dietary fiber; TDF: total dietary fiber; TE: Trolox equivalents; TSPC, total soluble phenolic compounds.

**Table 5:** Bioactive compounds and mineral bioaccessibility in control (C) and optimized pasta (oPQC, and oPKC) cooked to its optimal cooking time.

	C	oPQC	oPKC
Undigested			
GABA (mg/100 g)	4.94 ± 0.29 ^cA^	8.61 ± 0.16 ^bA^	22.11 ± 0.44 ^aA^
TSPC (mg GAE/100 g)	67.37 ± 6.04 ^cA^	146.46 ± 2.38 ^bB^	186.46 ± 10.75 ^aB^
ORAC (µmol TE/g)	1.33 ± 0.51 ^cC^	12.92 ± 0.34 ^bC^	15.99 ± 2.90 ^aC^
Gastric phase			
GABA (mg/100 g)	3.97 ± 0.36 ^cA^	7.21 ± 0.13 ^bAB^	18.70 ± 0.56 ^aB^
TSPC (mg GAE/100 g)	27.77 ± 16.8 b^B^	134.38 ± 6.28 ^aC^	139.58 ± 22.63 ^aC^
ORAC (µmol TE/g)	21.73 ± 1.74 ^bB^	25.54 ± 0.67 ^aB^	26.45 ± 1.69 ^aB^
Intestinal Phase			
GABA (mg/100 g)	5.12 ± 0.27 ^bA^	6.69 ± 0.55 ^bB^	16.26 ± 0.69 ^aB^
TSPC (mg GAE/100 g)	70.89 ± 4.61 ^bA^	261.86 ± 11.94 ^aA^	257.72 ± 14.59 ^aA^
ORAC (µmol TE/g)	142.34 ± 4.92 ^bA^	171.06 ± 9.53 ^aA^	164.93 ± 22.74 ^aA^
Soluble Fe (mg/100 g)	2.13 ± 0.09 ^c^	2.76 ± 0.14 ^a^	2.52 ± 0.03 ^b^
Fe bioaccessibility (%)	8.20 ± 0.35 ^a^	7.52 ± 0.09 ^b^	7.35 ± 0.11 ^b^
Soluble Ca (mg/100 g)	16.81 ± 0.74 ^c^	93.50 ± 3.77 ^a^	58.40 ± 1.07 ^b^
Ca bioaccessibility (%)	66.77 ± 2.95 ^a^	40.32 ± 1.62 ^b^	24.22 ± 0.45 ^c^

Data are means ± standard deviation (*n* = 2). Different lowercase letters denote statistical differences within rows (ANOVA, Bonferroni post hoc test, *p* < 0.05). Different uppercase letters denote statistical differences within columns (ANOVA, Bonferroni post hoc test, *p* ≤ 0.05). Abbreviations: C, control pasta formulated with 100% refined wheat flour; GABA, γ-aminobutyric acid; GAE, gallic acid equivalents; ORAC, oxygen radical absorbance capacity; oPKC, optimized wheat-based pasta supplemented with 13% sprouted kiwicha and 8% cushuro flour; oPQC, optimized wheat-based pasta supplemented with 15% sprouted quinoa and 15% cushuro flour; TE: Trolox equivalents; TSPC, total soluble phenolic compounds.

## Data Availability

Data are contained within the article.

## Supplementary Materials

The following supporting information ca be downloaded at https://www.mdpi.com/article/10.3390/foods12244395/s1. Table S1. Central composite design of the composite flour formulation used in pasta-making; Table S2. Effect of WF substitution ratio with sprouted pseudocereal and cushuro flours on PA, GABA, TSPC, and antioxidant activity (as determined by ORAC assay) of pasta; Figure S1. Images of pasta made with wheat, sprouted quinoa, and cushuro flour (PQC) using the different supplementation ratios from the experimental mixture design; Figure S2. Images of pasta made with wheat, sprouted kiwicha and cushuro flour (PKC) using the different supplementation ratios from the experimental mixture design.
