# Supplementary material for: Enhancing Nutritional Profile of Pasta: The Impact of Sprouted Pseudocereals and Cushuro on Digestibility and Health Potential
Source: Foods. 2023 Dec 6;12(24):4395. doi: 10.3390/foods12244395 (PMC10742926; doi:10.3390/foods12244395)

Enhancing Nutritional Profile of Pasta: Impact of Sprouted Pseudocereals and Cushuro on Digestibility and Health Potential

Luz María Paucar-Menacho [1], Juan Carlos Vásquez Guzmán [1], Wilson Daniel Simpalo-López [1], Williams Esteward Castillo-Martínez [1], and Cristina Martínez-Villaluenga [2,*]

Table S1. *Central Composite Design of the composite flour formulation used in pasta-making.*

Recipe No.	*Code levels*			*Levels*		
	A	*B*	*C*	*WF*	*SKF / SQF*	*CuF*
1	0	1	1	70	15	15
2	1	0	1	80	5	15
3	1	1	0	80	15	5
4	0.5	0.5	1	75	10	15
5	0.5	1	0.5	75	15	10
6	1	0.5	0.5	80	10	10
7	0.67	0.33	0.33	76	12	12
8	0.33	0.33	0.33	74	13	13
9	0.83	0.83	0.33	79	8	13
10	0.83	0.33	0.83	79	13	8
11	1	1	0	80	15	5
12	1	0.5	0.5	80	10	10
13	0	1	1	70	15	15
14	1	0	1	80	5	15

Abbreviations: CuF: cushuro flour; SKF: sprouted kiwicha flour; SQF: sprouted quinoa flour; WF: wheat flour.

Table S2. *Effect of WF substitution ratio with sprouted pseudocereal and cushuro flours on PA, GABA, TSPC, and antioxidant activity (as determined by ORAC assay) of pasta.*

Pasta Type	*Recipe No.*	*Flour Ratio*			*PA (g/100 g dw)*	*GABA (mg/100 g dw)*	*TSPC (mg GAE/100 g dw)*	*ORAC (µmol TE/g dw)*
		WF (A)	*SQF/SKF (B)*	*CuF (C)*				
PQC	1	70	15	15	0.23±0.01	8.67±0.58	169.30±15.52	16.70±1.39
	2	80	5	15	0.16±0.03	8.13±0.02	107.34±6.77	13.35±1.32
	3	80	15	5	0.25±0.01	22.78±1.35	163.18±9.96	21.64±1.15
	4	75	10	15	0.19±0.01	9.42±0.34	142.76±4.27	17.33±1.01
	5	75	15	10	0.22±0.01	5.35±5.71	146.15±7.04	22.90±1.17
	6	80	10	10	0.20±0.03	12.40±0.80	122.77±5.27	16.92±1.28
	7	76	12	12	0.16±0.02	9.71±0.48	140.11±5.90	17.15±1.17
	8	74	13	13	0.16±0.03	10.19±0.19	150.15±9.80	14.86±1.11
	9	79	8	13	0.14±0.03	8.37±0.09	127.07±10.42	14.03±1.33
	10	79	13	8	0.27±0.01	17.70±0.19	158.94±7.98	22.10±0.73
	11	80	15	5	0.25±0.01	22.78±1.35	163.18±9.96	21.64±1.15
	12	80	10	10	0.20±0.03	12.40±0.80	122.77±5.27	16.92±1.28
	13	70	15	15	0.23±0.01	8.67±0.58	169.30±15.52	16.70±1.39
	14	80	5	15	0.16±0.03	8.13±0.02	107.34±6.77	13.35±1.32
PKC	1	70	15	15	0.06±0.01	37.87±0.65	227.06±11.53	29.79±2.87
	2	80	5	15	0.04±0.00	4.11±1.59	191.88±14.11	21.42±2.48
	3	80	15	5	0.06±0.01	44.59±2.42	174.56±9.22	36.47±0.77
	4	75	10	15	0.07±0.01	36.43±1.74	202.89±10.10	25.48±1.61
	5	75	15	10	0.09±0.01	46.46±1.05	196.38±10.60	31.79±1.36
	6	80	10	10	0.09±0.02	30.45±2.58	165.74±8.25	26.47±1.54
	7	76	12	12	0.08±0.01	33.14±0.32	175.01±7.93	24.58±1.74
	8	74	13	13	0.05±0.01	34.97±0.41	170.70±8.35	27.25±1.15
	9	79	8	13	0.05±.01	30.92±2.34	143.12±11.42	21.83±2.19
	10	79	13	8	0.09±0.01	38.80±1.84	147.54±12.42	24.27±2.09
	11	80	15	5	0.06±0.01	44.59±2.42	174.56±9.22	36.47±0.77
	12	80	10	10	0.09±0.02	30.45±2.58	165.74±8.25	26.47±1.54
	13	70	15	15	0.06±0.01	37.87±0.65	227.06±11.53	29.79±2.87
	14	80	5	15	0.04±0.00	4.11±1.59	191.88±14.11	21.42±2.48

Data are means ± standard deviation (n = 2). Abbreviations: GABA, γ-aminobutyric acid; GAE, gallic acid equivalents; ORAC, oxygen radical absorbance capacity; PA, phytic acid; PKC, wheat-based pasta supplemented with sprouted kiwicha and cushuro flour; PQC, wheat-based pasta supplemented with sprouted quinoa and cushuro flour; TSPC, total soluble phenolic compounds; TE: Trolox equivalents.

Figure S1. *Images of pasta made with wheat, sprouted quinoa and cushuro flour (PQC) using the different supplementation ratios from the experimental mixture design*

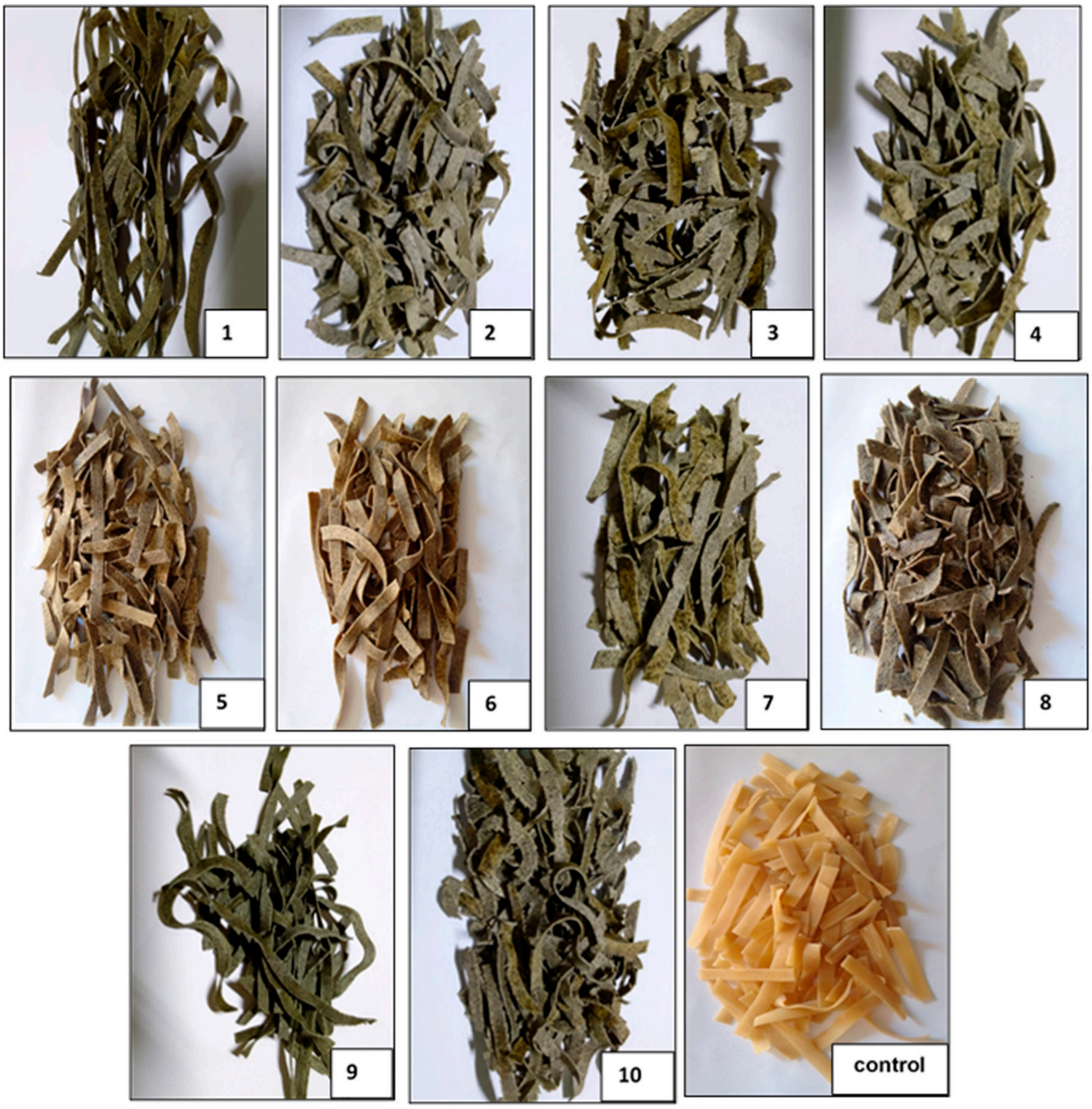

Figure S2. *Images of pasta made with wheat, sprouted kiwicha and cushuro flour (PKC) using the different supplementation ratios from the experimental mixture design*

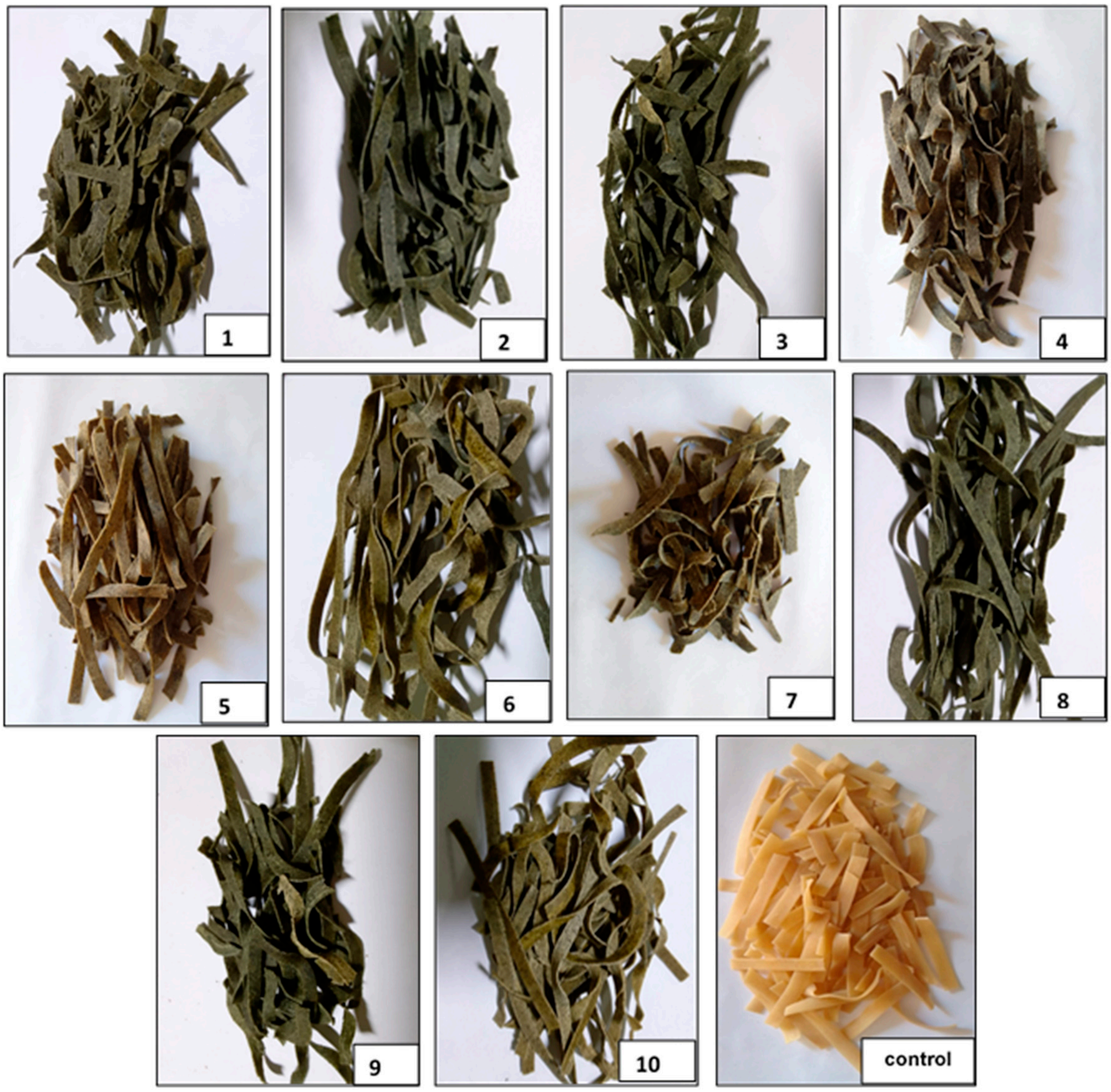

Supplement: Supplementary file 1 [file foods-12-04395-s001.zip › foods-2742006-supplementary.pdf]
